# Brain mineralocorticoid receptor activation and antagonism in heart failure with preserved ejection fraction: a hypothesis

**DOI:** 10.1093/eurheartj/ehag450

**Published:** 2026-07-02

**Authors:** Lars H Lund, Bertram Pitt

**Affiliations:** Department of Medicine, Karolinska Institutet, S1:02, Stockholm 171 76, Sweden; Department of Cardiology, Karolinska University Hospital, S1:02, Stockholm 171 76, Sweden; Division of Cardiovascular Medicine, Department of Internal Medicine, University of Michigan, Ann Arbor, MI, USA

## Abstract

**Graphical Abstract:**
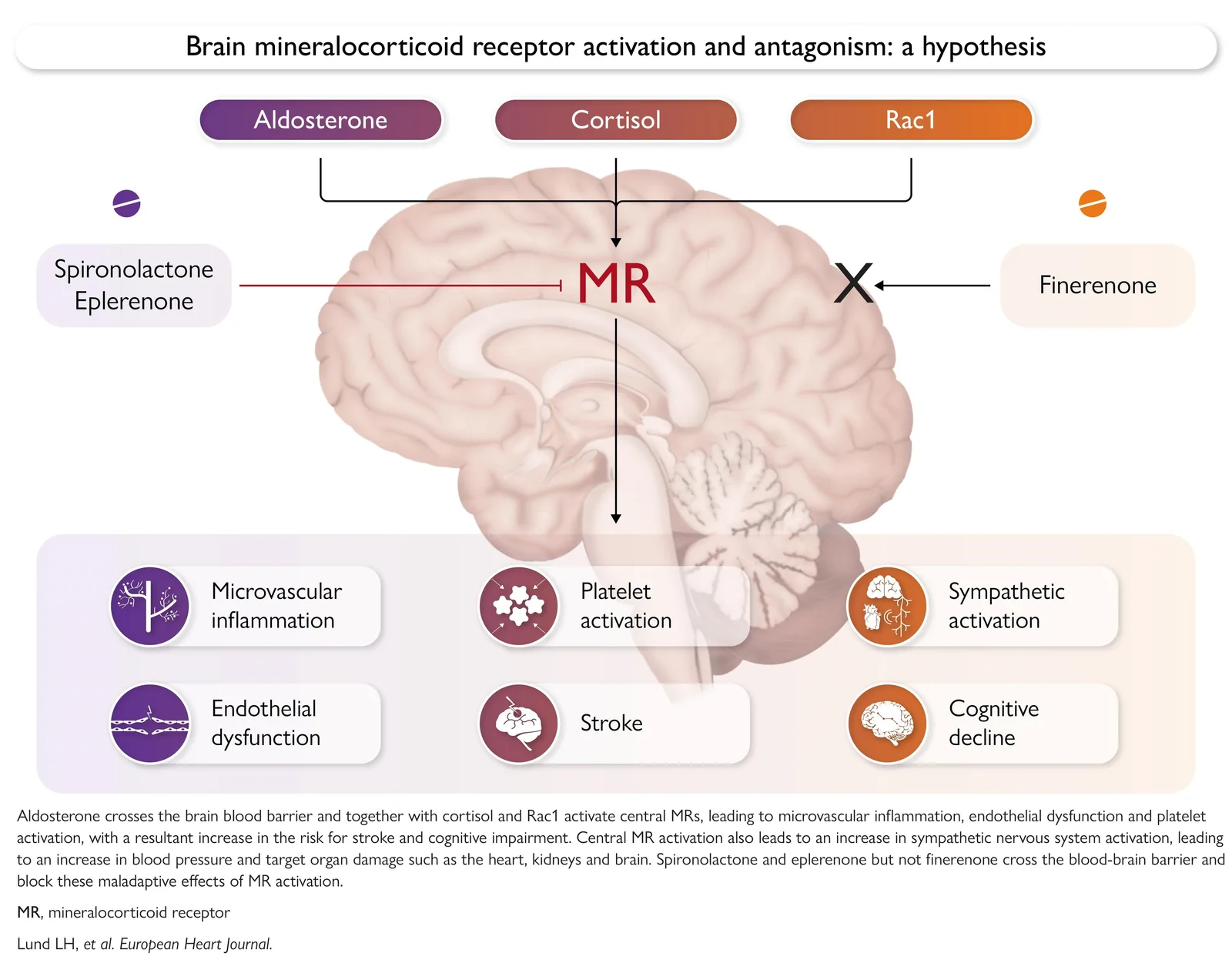
Image generated by BioRender.

Image generated by BioRender.

## Steroidal and non-steroidal mineralocorticoid receptor antagonists

Aldosterone is elevated in cardiovascular–renal–metabolic conditions, such as heart failure (HF), vascular disease and endothelial dysfunction, chronic kidney disease (CKD), Type 2 diabetes, and visceral obesity, as well as primary (low renin) aldosteronism. Aldosterone is also implicated in causing and/or exacerbating these conditions.

The steroidal mineralocorticoid receptor antagonist (MRA) spironolactone is indicated for HF with reduced ejection fraction and for treatment-refractory hypertension. In the TOPCAT trial in HF with preserved ejection fraction (HFpEF), spironolactone was neutral overall but effective in the Americas. This *post hoc* analysis is hypothesis generating only and thus serves as a rationale for the soon to report HFpEF trials SPIRRIT-HFpEF (NCT02901184) and SPIRIT-HF (NCT04727073). In the BARACK-D trial in CKD Stage 3b, spironolactone was discontinued in two-thirds of patients due to worsening estimated glomerular filtration rate (eGFR), side effects, and/or hyperkalaemia, and randomized assignment to spironolactone did not reduce cardiovascular outcomes,^1^ possibly due to the large number of patients who prematurely discontinued spironolactone. In contrast, in diabetic kidney disease^2,3^ and HFpEF,^4^ the non-steroidal MRA (nsMRA) finerenone was effective in reducing the composite kidney endpoint as well as HF hospitalizations and the composite of HF hospitalizations and cardiovascular death.

Thus, which is the MRA of choice for HFpEF? There are several considerations. Apart from the lower cost, we raise the hypothesis that there are mechanistic reasons relating to the maladaptive effects of mineralocorticoid receptor (MR) activation in the central nervous system (CNS) and the ability of spironolactone but not finerenone to cross the blood–brain barrier (BBB).

## Heart failure with preserved ejection fraction, aldosterone, and the central nervous system

Patients with HFpEF are often elderly, with frequent comorbidities associated with increased adrenal aldosterone synthesis and an increase in plasma aldosterone concentration. Hypertension, often with concomitant primary aldosteronism, CKD, and visceral obesity, contributes to increased aldosterone production. These conditions are associated with increased 11 beta-hydroxysteroid dehydrogenase type 1 (11β-HSD1) in the vasculature and decreased 11β-HSD2 activity in the kidneys, leading to activation of the MR also by cortisol and Rac-1.^5,6^ These comorbidities are also associated with an increase in BBB permeability.^7^ In the elderly, there is also a decrease in the P-glycoprotein transport system that normally extrudes aldosterone from the CNS.^8^ These factors contribute to an increase in aldosterone crossing the BBB and activation of CNS MRs, which are expressed in the microvasculature, hippocampus, and amygdala.^9^ In patients with HFpEF, especially the elderly, the CNS MRs are further activated by cortisol and Rac-1.^10^ Activation of CNS MRs has been associated with an increase in microvascular inflammation, endothelial dysfunction, and platelet activation, with a resultant increase in the risk for stroke and cognitive impairment.^11^ Furthermore, an increase in CNS MR activation has been associated with an increase in sympathetic nervous system activation, leading to an increase in blood pressure and target organ damage, such as the heart, kidneys, and brain. Thus, a relatively large percentage of patients with HFpEF can be expected to have an increase in BBB permeability and activation of CNS MRs with an increased risk of treatment-resistant hypertension, stroke, and cognitive impairment. These observations are of importance in considering the relative effectiveness of finerenone and spironolactone in HFpEF.

## Mineralocorticoid receptor antagonists and the blood–brain barrier

Spironolactone and its metabolite canrenone have been shown to cross the BBB^12^ and to antagonize the MR whether activated by aldosterone, cortisol, or Rac-1. In contrast, the nsMRA finerenone has not been shown to cross the BBB^13^ and thus would not be expected to counter the effects of CNS MR activation. Specifically, finerenone has low lipophilicity and high polarity, resulting in restricted BBB penetration, as demonstrated by low CNS distributions of radiolabelled finerenone.^13,14^ Furthermore, the reactive increase in aldosterone associated with the administration of an MRA could potentially increase the concentration of aldosterone available to cross the BBB and enter the CNS and unopposed increase the risk of stroke and cognitive impairment. This increase in CNS aldosterone activity would be expected to be blocked by spironolactone but not finerenone. Indeed, in patients with hypertension and/or HF, there is suggestive evidence that spironolactone can reduce the risk of stroke, and preclinical studies suggest that it may reduce cognitive impairment.^15^ However, it is unknown whether this is merely an effect of lowering blood pressure through peripheral effects, or an additional effect involving central MRs. Both spironolactone and finerenone lower blood pressure, albeit to a modest extent, and any incremental risk reduction of spironolactone vs finerenone would require distinct differences regarding our hypothesized role of aldosterone and MRs in the CNS. Furthermore, we cannot rule out that increased BBB permeability among patients with HFpEF, mostly elderly, may also facilitate finerenone entering the CNS, but whatever the baseline permeability is, the differences between spironolactone and finerenone, if clinically relevant, would be additive. We are not aware of any data demonstrating an effect of finerenone on stroke or cognitive impairment, and in FIDELITY there was no effect of finerenone on stroke,^3^ but this would be an important area to study in *post hoc* analyses of completed and in future finerenone and spironolactone studies.

In conclusion, these are hypothetical mechanistic reasons to consider spironolactone (or where available canrenone) as potentially more effective than nsMRAs regarding stroke and cognitive impairment in patients with HFpEF. This hypothetical benefit needs to be weighed against the definitive FINEARTS-HF results vs *post hoc* TOPCAT results, and importantly, the apparent better tolerability of finerenone vs spironolactone, with lesser gynecomastia, sexual dysfunction, and hyperkalaemia. Trials with direct comparisons between different agents or classes of drugs targeting aldosterone are unlikely to be sponsored by industry. However, previous, ongoing, and future trials should examine in detail the risk of and potential protection against cognitive impairment and stroke before reaching a decision as to the optimal MRA in HFpEF. Given the increase in the elderly population and the large cost of caring for stroke and cognitive impairment, it may be cost-effective for public funders to sponsor direct comparisons of different agents targeting aldosterone.
